# Whole-genome haplotyping approaches and genomic medicine

**DOI:** 10.1186/s13073-014-0073-7

**Published:** 2014-09-25

**Authors:** Gustavo Glusman, Hannah C Cox, Jared C Roach

**Affiliations:** Institute for Systems Biology, Terry Avenue North, Seattle, WA 98109 USA

## Abstract

Genomic information reported as haplotypes rather than genotypes will be increasingly important for personalized medicine. Current technologies generate diploid sequence data that is rarely resolved into its constituent haplotypes. Furthermore, paradigms for thinking about genomic information are based on interpreting genotypes rather than haplotypes. Nevertheless, haplotypes have historically been useful in contexts ranging from population genetics to disease-gene mapping efforts. The main approaches for phasing genomic sequence data are molecular haplotyping, genetic haplotyping, and population-based inference. Long-read sequencing technologies are enabling longer molecular haplotypes, and decreases in the cost of whole-genome sequencing are enabling the sequencing of whole-chromosome genetic haplotypes. Hybrid approaches combining high-throughput short-read assembly with strategic approaches that enable physical or virtual binning of reads into haplotypes are enabling multi-gene haplotypes to be generated from single individuals. These techniques can be further combined with genetic and population approaches. Here, we review advances in whole-genome haplotyping approaches and discuss the importance of haplotypes for genomic medicine. Clinical applications include diagnosis by recognition of compound heterozygosity and by phasing regulatory variation to coding variation. Haplotypes, which are more specific than less complex variants such as single nucleotide variants, also have applications in prognostics and diagnostics, in the analysis of tumors, and in typing tissue for transplantation. Future advances will include technological innovations, the application of standard metrics for evaluating haplotype quality, and the development of databases that link haplotypes to disease.

## Introduction

Technological progress has enabled the routine resequencing of human genomes. These genomes include rare variants at high frequency [1,2] that are the result of exponential human population growth over the past hundred generations [3]. These variants can affect single nucleotides or larger genomic ranges by substitution, insertion, deletion, or change in copy number. Combinations of variants are present in *cis* on the same physical molecule or in *trans* on homologous chromosomes. This set of *cis* and *trans* relationships between the variants, known as the phase of the variants, affects the interpretation and implications of the relationships between genotypes and phenotypes, including disease phenotypes [4-6]. To simplify the discussion, we define a haplotype in general terms as a contiguous subset of the information contained in a molecule of DNA (Box 1)*.* An example of a haplotype by this definition, grounded on molecular observation, is the actual sequence inherited from one parent and spanning one or more genes in a specific genomic region of interest. A corollary of this definition is that the longest possible haplotype, the ‘chromosome haplotype’, is the full sequence of a chromosome that an individual inherited from one parent. Haplotypes have a number of important roles and applications that are listed in Box 2.

Analysis of haplotypes falls generally into three categories: 1) elucidating the ‘haplotype block’ structure of the genome, 2) employing haplotypes as genetic markers, and 3) understanding haplotypes as functional units [7]. As the number of observable genetic variants increases, so does the number of observable haplotypes. This increase in observed variants is largely the result of rare variants assayed by whole-genome sequencing (WGS). As a result, there are now many observed haplotypes with frequencies too small to estimate using population-based inference methods. Recent technological advances have enabled the determination of haplotypes by direct observation of molecular data and from genetic information, and with decreased reliance on population-based statistical estimation.

Historically, it has been difficult to distinguish between the homologous haplotypes of autosomes inherited from each of the parents. For that reason, allele information from each pair of autosomal chromosomes is typically comingled into one sequence of information: the unphased genotype sequence. Phasing (or haplotyping) describes the process of determining haplotypes from the genotype data. Until recently, cost, lack of data, and computational intractability has limited the availability of phased whole-genome haplotypes [4].

**Figure 1:**
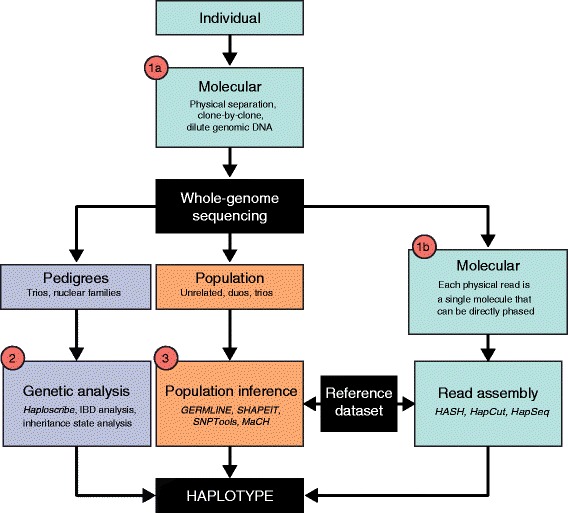
**Overview of the three methods for phasing whole-genome sequence data.** Phasing is achieved by (1) molecular methods, (2) genetic analysis, or (3) population inference. Molecular methods focus on individual samples and involve either (a) processing genomic DNA prior to sequencing or (b) leveraging the single-molecule characteristic of each physical read. Genetic analysis and population inference process sequenced genomes from pedigrees and population cohorts, respectively.

**Figure 2:**
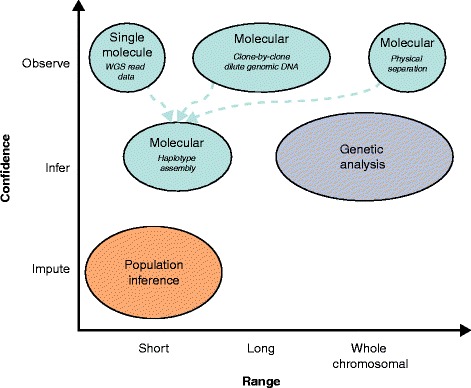
**The properties of phasing methods.** The level of confidence in phasing and the achievable range of phased sequence length both vary depending on the method used. Molecular methods provide direct observations from single molecules and therefore the level of confidence in the results is high. The phased sequence length that can be achieved by these methods has a wide range, which depends on the method employed. Molecular observations can be assembled into haplotypes (dashed arrows), adding moderately to the range of phased sequence length, and potentially introducing inference error. Genetic analyses infer phase by leveraging the property of Mendelian segregation and can phase entire chromosomes. Population inference methods are probabilistic and limited to the generation of short-range haplotype blocks.

There are three basic methods for phasing: molecular, genetic, and population analysis. Molecular haplotyping is rooted in the analysis of single molecules (Figures 1 and 2, Table 1). If the molecule haplotyped is shorter than a chromosome, molecular haplotyping can be followed by haplotype assembly. Increasingly clever methods are being deployed to exploit high-throughput parallelization, combining data from measurements of many single molecules to build longer haplotypes. Genetic haplotyping requires pedigrees and can yield chromosome-length haplotypes [8]. Population haplotyping requires models of the population structure of haplotypes and can only phase common variations. These three approaches can also be combined to create hybrid strategies. As a general rule, these methods can be used to phase any combination of single nucleotide variants (SNVs; commonly called single nucleotide polymorphisms (SNPs) when they are frequent enough in the population), insertions and deletions (indels), and copy number variants (CNVs). SNVs and short indels are typically easier to phase because they can be observed within individual sequence reads. Larger variants, such as CNVs, are typically assessed using genotyping arrays [9,10].

**Table 1:** **Overview of whole-genome haplotyping methods**

**Method**		**Minimal cohort**	**Advantages**	Limitations*
Molecular	Single and paired-end physical reads	Individual	Haplotype is directly observed from sequence data	Produces short haplotypes, even after assembly
			Simple	
			Can resolve private and rare haplotypes	
			Can phase *de novo* variants	
	Chromosome sorting, clone-by-clone, dilution, proximity ligation	Individual	Haplotype is directly observed from sequence data	May be labor intensive, time-consuming and expensive, therefore
			Highly accurate	difficult to translate to large sample sizes
			Can resolve private and rare haplotypes	
			Can phase *de novo* variants	
			Can resolve long-range and chromosome-length haplotypes (depending on method)	
			Ideal for generating personalized genome-resolved haplotypes	
	Haplotype assembly	Individual	Leverages molecular haplotype information from WGS data and/or from sorted chromosomes, clones	Assembly requires variants in overlapping sequence reads
			Works well when molecular haplotypes are long (that is, from cosmid or BAC)	Limited by the accuracy and availability of suitable reference data
				Generate short-range haplotypes
				May introduce phase errors
Genetic analysis		Trios, nuclear families	Can accurately phase high-throughput short-read sequencing reads	Cannot resolve sites where all family members are heterozygous
			Low error rate	
			Precisely maps recombinations and inheritance states	May not be possible to ascertain family members
			Enables detection of sequencing errors	
			Can phase private and rare alleles	
			Can phase entire chromosomes	
			Suitable for clinical applications	
Population inference		Unrelated individuals, duos, trios	Cost-effective	Can only phase common variants
			Facilitates haplotype imputation in samples with low-density microarray panels	Difficult to impute private variants or rare haplotypes
			Useful when family members cannot be ascertained	Limited by the accuracy and availability of suitable reference data
			Large sample sizes increase accuracy	Generates short-range haplotypes
			Good for large samples of unrelated individuals	Sample size impacts haplotype frequency estimations
			Incorporation of family duos and trios improves accuracy	Methods are probabilistic and accuracy must be balanced against computational costs

*All of these methods are limited by the accuracy of the sequence data.

In this review, we describe in detail the three main methodologies for phasing variants and their integration into combination strategies, and we provide quality metrics (Box 3). Finally, we provide an overview of the applications of whole-genome haplotyping in genomic medicine.

## Molecular haplotyping

Molecular haplotyping involves the direct observation of alleles on a single molecule. These molecules are often single sequence reads, ranging in size from tens of bases to thousands of bases. When two variants are observed in the same physical read, or in paired reads derived from the same molecule, they can be directly phased. Therefore, same-read molecular haplotyping gains power with sequencing read length, and the major source of error is the sequencing error rate. Although often overlooked because of its simplicity, sequencing is the most common form of molecular haplotyping. Other forms of molecular haplotyping include restriction fragment analysis, optical mapping, and coded fluorescence hybridization approaches [11].

Long-range binning can be achieved by physical separation of the two haploid genomes prior to sequencing. Binning methods are able to resolve private and rare haplotypes and can be used to generate personalized genome-resolved haplotypes. Sequencing isolated sperm cell genomes [12] is one simple approach, but applicable only to males. Chromosome isolation methods do not require sequencing coverage to the depth needed to resolve possibly heterozygous positions [10]. Whole-chromosome sorting methods include microdissection, fluorescence-activated cell sorting (FACS) and microfluidics. Chromosomes are individually tagged or separated into pools that tend to contain at most one copy of a chromosome. These are genotyped or sequenced to generate whole-chromosome haplotypes. Microdissection involves arresting cells in metaphase and spreading the chromosomes to isolate them [13]. FACS separates individual chromosomes, which are then amplified and tagged before sequencing [14]. The ‘direct deterministic phasing’ method uses microfluidic devices to capture a single metaphase cell and partition each intact chromosome [10].

Semiconductor-based nanotechnologies are being applied to assay single DNA molecules, deriving very long-range haplotype information. NabSys (Providence, RI, USA) tags DNA molecules with probes that are specific to particular chromosomal locations and passes single molecules of DNA with bound tags through solid-state nanodetectors to identify the locations of bound tags [15]. BioNano Genomics (San Diego, CA, USA) labels DNA using nicking endonucleases and fluorescently labeled nucleotides, then visualizes single molecules in linearized nanochannels [16]. Both technologies yield *de novo* genome-wide maps, informing structural variation and haplotypes. Nevertheless, no current technology captures all variants in the genome; for example, most are unable to assay trinucleotide repeats. These technologies are changing rapidly, and Buermans and den Dunnen [17] have provided a recent review of the types of variants assayed by some of these technologies. The principles and methods of haplotyping described here will apply even as methods change. In some cases, combining a technology that assays large variants (for example, BioNano) with one that assays SNPs (for example, pairwise end sequencing) may best address a particular need.

## Haplotype assembly

Any set of two or more overlapping haplotypes can be assembled into a single haplotype. Typically, after generation of many individual molecular haplotypes, sequence assembly is used to identify overlapping sequences and thus to infer a longer haplotype [18-20]. The haplotypes being assembled may be derived from heterogeneous data sources but haplotype assembly is most commonly based on a set of molecular haplotypes [21-23], so we will discuss this prior to the discussion of genetic and population-inferred haplotypes.

Assembly of molecular sequences from fragments predates the ability to sequence DNA. Assembly was originally employed for determining the sequence of proteins [24]. Before the Human Genome Project (HGP), genome maps were assembled from restriction-fragment haplotypes [25]. During the HGP, haplotype reconstruction relied on the assembly of matched-end sequences of clones. As the HGP wound down, for economy of scale, there was a general shift away from long-read towards short-read sequencing. This shift increased the difficulty of haplotype assembly directly from shotgun reads, and resulted in a revival in algorithms for haplotype assembly. Lancia *et al*. [26] describe the ‘SNPs haplotyping problem’ by looking at the fundamental constraint shared by the group of algorithms that solve this problem: that all sequence reads must be partitioned into exactly two bins. These algorithms generally allow for sets of raw reads to be constrained to co-occur in the same bin. Such constraints arise either from paired end data or from pooling strategies. Clever experimental designs have maximized the utility of these constraints, particularly those that use statistical or molecular techniques to bin reads from a particular haplotype.

In 2007, Levy *et al.* [27] used single sequence reads together with some mate pairs to build long-range haplotypes for an individual genome, with haplotypes reaching several hundred kilobases. In 2009, McKernan *et al*. [28] used a ligation-based sequencing assay to phase a single genome physically into blocks averaging several kilobases. In 2011, Kitzman *et al*. [29] produced 350 kb haplotype blocks by subpooling a fosmid library. Suk *et al*. [30] also used fosmid pool-based sequencing to assemble variants into haplotypes of approximately 1 Mb, up to a maximum length of 6.3 Mb; fosmids were tiled into contiguous molecular haplotype sequences based on sequence overlaps [31]. In 2013, Kaper *et al*. [32] also used a dilution, amplification and sequencing approach to compile haplotypes of several hundred kilobases in length.

Extreme dilution of genomic DNA can generate long-range haplotypes without requiring the sorting of metaphase chromosomes or cloning. These methods recreate, with twists, the basic method used to sequence the human genome: local haplotypes (in the order of tens of kilobases) are first carefully sequenced and then strung together by aligning overlaps. Dilution methodologies allow long fragments to be shotgun sequenced with short reads [18]. If these long fragments overlap with a sufficient fingerprint [33], then haplotypes up to 1 Mb may be achieved by chromosomal walking [34]. The number of DNA molecules in a pool is small enough that there is little chance that repeated or duplicate sequences will occur within a pool. Therefore, DNA dilution methods simplify both *de novo* assembly and mapping reads to a reference genome. Nevertheless, these methods can be confounded by the local presence of repetitive sequences. Commercialization of dilution methodologies now includes Complete Genomics’ ‘long fragment read’ (LFR) [35] and Illumina’s Moleculo technology [36]. For LFR, long parental DNA fragments are separated into distinct pools and sequenced using pairwise end sequencing. Moleculo implements statistically aided, long-read haplotyping (SLRH) by further phasing initial contigs with population information using the *Prism* software (Table 2).

**Table 2:** **Summary of selected software available for whole-genome haplotyping**

**Method**	**Software**	**Summary**	**Reference(s)**
Molecular - haplotype assembly	*HapCut* (OSS)	A combinatorial approach implementing a max-cut-based algorithm and optimized minimum error correction (MEC) solution	[22]
	*Single Individual Haplotyper* (OSS)	A collection of algorithms including *RefHap*, a heuristic algorithm for sorting reads into haplotype bins	[31,37]
	*H-BOP* (OSS)	Heuristic algorithm for optimizing a combination of the MEC and Maximum Fragments Cut models	[38]
	*MixSIH* (OSS)	Probabilistic mixture model	[39]
	*HASH* (OSS)	Markov chain Monte Carlo algorithm	[21]
Genetic analysis	*Haploscribe* (OSS)	Implements a parsimony approach to generate inheritance state vectors and a hidden Markov model to deduce haplotypes	[8]
Population inference	*Beagle* (OSS)	Phased input data are used to build a local haplotype cluster model, which is sampled using a hidden Markov model. Iterations and the Viterbi algorithm are used to select the ‘most likely’ haplotype	[40]
	*fastPHASE* (OSS)	Enhancement of *PHASE*. Implements a haplotype-clustering model with a fixed number of clusters and hidden Markov model assumptions for cluster membership. Expectation-maximization methods are incorporated for parameterization	[41]
	*GERMLINE* (OSS)	Implements a hashing-algorithm approach to identifying whole-haplotype segment sharing	[42]
	*IMPUTE2*	Pre-phasing, imputation and haplotype sampling strategy incorporating a Monte Carlo algorithm and Markov model calculations	[43]
	*MaCH*	Implements a Markov Chain algorithm for genotype imputation and haplotyping	[44]
	*PHASE* (OSS)	Implements Bayesian haplotype reconstruction	[45]
	*SHAPEIT*	Implements hidden Markov model sampling	[46,47]
	*SNPTools* (OSS)	A population imputation pipeline that generates genotype likelihoods using a binary sequence map-specific binomial mixture model. Haplotypes are then sampled using a hidden Markov model	[48]
	*WinHAP* (OSS)	Scalable sliding windows are used to optimize haplotypes and a parsimony approach iteratively restricts the number of solutions	[49]
Combination strategies	*HARSH* (OSS)	Sampling within a probabilistic model combining read data with a reference panel of haplotypes. Successor to *Hap-SeqX*	[50]
	*SHAPEIT2*	Adds short-read molecular information to population inference	[51]
	*Prism*	Combines haplotype assembly and population inference	[36]
	*PPHS*	Implements a phylogeny model to estimate haplotype frequencies recursively using the expectation maximization algorithm	[52]
	*FamilyQuartet* (OSS)	Integrates physical, genetic and population phasing	[53]

*Abbreviations:* *OSS* open source software, *MEC* minimum error correction.

Several algorithms exist to assemble reads into haplotypes (Table 2). *HASH* (haplotype assembly for single human) uses a Markov chain Monte Carlo (MCMC) algorithm and graph partitioning approach to assemble haplotypes given a list of heterozygous variants and a set of shotgun sequence reads mapped to a reference genome assembly [21]. *HapCut* uses the overlapping structure of the fragment matrix and max-cut computations to find the optimum minimum error correction (MEC) solution for haplotype assembly [22]. There are many other sequence assembly algorithms, reviewed elsewhere [54,55]. Duitama *et al*. [31] reviewed eight algorithms for the ‘SNPs haplotyping problem’ with binned reads as input. They concluded that, under a reasonable compromise between accuracy, completeness, and computational resources, *ReFHap* (*Reliable and Fast Haplotyping*) [37] yields the best results for a low-coverage fosmid pooling approach, which they term single individual haplotyping (SIH). More recent algorithms claim improvements on parameters such as speed and accuracy (for example, *H-BOP* [38]) or focus on improving performance in the presence of high error rates [39,56]. *MixSH* shows good performance as evaluated by pair consistency, a version of a metric described in Box 3 [39].

The process of assembly may introduce phase errors at the joins between component haplotypes, and so should best be done when the overlaps between fragments can be inferred with high confidence. Such confidence can be gained both by identification of unique overlapping fingerprints or by physical separation of the original molecules. Haplotype assembly has worked very well when the underlying haplotypes are long, such as those determined by sequencing a clonal source such as a cosmid or bacterial artificial chromosome (BAC) [14,29]. We therefore expect to see increasing development of technologies that generate sequence reads in the range of many thousands of bases to facilitate haplotype assembly. These long sequences will be generated by strobe sequencing, nanopore sequencing, and perhaps other technologies [57,58].

The existence of chromosome territories in the nucleus can also be exploited for long-range haplotyping. In an innovative approach, pairs of reads that are likely to come from the same haplotype are generated by cross-linking chromatin sites that are potentially distant along a chromosome but spatially close within the nucleus. This technique is known as ‘Hi-C’, and was simultaneously exploited by three different groups for sequence assembly [59-61]. Selvaraj *et al*. [62] focused on haplotyping using Hi-C (which they term ‘HaploSeq’), and in their initial report using low coverage sequencing they phased approximately 81% of sequenced alleles.

Disparate sources of haplotyping information and markers can also be assembled. For example, the 1000 Genomes Project Consortium recently produced an integrated haplotype map of SNPs, small indels and larger deletions derived from SNP arrays or from exome and whole-genome sequencing [63].

## Genetic analysis

The principles of Mendelian segregation of alleles in pedigrees can be used to deduce the phasing of variants observed in ordered genotypes. At the simplest level of a family trio (both parents and one child), very simple rules indicate which alleles in the child were inherited from each parent, thus largely separating the two haplotypes in the child. The remaining (not inherited) parental haplotypes can then be reconstructed using a simple exclusion rule. As the locations of recombinations are not known, the inferred parental haplotypes will have a phase error at each recombination. These low-frequency phase errors (Box 1) will have little effect on short-range haplotypes but will scramble chromosomal haplotypes.

In the context of a family quartet (two full siblings and their parents), whole-genome sequences from high-throughput paired-end short-reads can generate complete chromosomal haplotypes for all family members [8,64]. The method can be extended to larger pedigrees by tiling or MCMC approaches [65]. Tiling can accumulate small errors with each tile, and so MCMC and similar approaches are likely to be the best methods for pedigrees spanning more than four generations. Haploscribe is a suite of software scripts that phase whole-genome data across entire chromosomes by genetic analysis [8]. Haploscribe implements a parsimony approach to generate meiosis-indicator (inheritance state) vectors and uses a hidden Markov model (HMM) to deduce haplotypes spanning entire chromosomes. These haplotypes are nearly 100% accurate and potentially suitable for medical diagnostics.

The rule-based nature of genetic phasing has a useful property: some family genotypes are not consistent with the expected patterns of inheritance, and are thus highlighted as probable sequencing errors or, rarely, as *de novo* mutations [64]. Mendelian inheritance errors (MIEs) are sites in which the genotype of a child is inconsistent with inheritance from one or both parents. In state consistency errors (SCEs), the genotype of each child is consistent with both parents but the combination of offspring genotypes is inconsistent with the prevailing inheritance state around that locus, as determined from neighboring sites.

Genetic analysis enables the phasing of rare alleles that cannot otherwise be accomplished by reference to population-based data. Phasing information obtained through the sequencing of the genomes of family members maps recombinations and inheritance states at high resolution, highlighting the regions of the genome where causal variants segregate. The resulting haplotypes are highly accurate and complete. Nevertheless, genetic analysis cannot phase positions in which all family members are heterozygous. Furthermore, it is not always feasible to recruit the required participants for family-based studies. In the absence of a family context, molecular haplotyping is an excellent choice because it does not require DNA samples from other family members. We predict that, in the next decade, molecular haplotyping will largely supplant the need for genetic analysis.

Dewey *et al*. [66] employed family inheritance-state analysis to control sequencing error and inform haplotype phasing to quantify genome-wide compound heterozygosity from high-throughput sequencing data. To define the inheritance states of neighboring SNVs in the family quartet, Dewey *et al*. first used a heuristic algorithm that binned allele assortments, followed by a HMM in which the hidden states corresponded to the four possible inheritance states in the quartet and the two error states described by Roach *et al*. [64]. A combination of pedigree data and statistical phasing based on inheritance state analysis was then used to infer phase for the majority of positions in each child’s genome. For uniformly heterozygous positions, the minor allele was assigned to the paternal and maternal chromosome scaffolds using pair-wise pre-computed population linkage disequilibrium (LD) data from the SNP Annotation and Proxy Search (SNAP) database [67]. These algorithms successfully determined genome-wide, long-ranging phased haplotypes in the family quartet. Phased variant data were also used to determine parental contribution to each child’s disease risk in the context of thrombophilia.

## Population inference

Population analysis leverages shared ancestry information to infer the most likely phasing of variants. The reference population can range from the very large (for example, the global human population), to the narrowly defined (for example, an isolated community). Because population relationships may be distant or cryptic, methodologies for population analysis are statistical and not deterministic. Also, because many more meioses separate all of the genomes in a large population, the length of haplotypes determined by population analysis is typically limited to thousands or tens of thousands of bases. Population inference methods work well on genotyping panels, which are compilations of common SNPs. As marker density increases, brute-force algorithms become less tractable, and algorithms such as those based on HMMs are employed [68]. Discerning private and rare haplotypes by population-based methods is highly challenging. Population analysis cannot phase *de novo* mutations, rare variants or structural variants. If a rare variant is assigned to a haplotype by other methods, however, its presence on a haplotype determined by common SNPs can be probabilistically inferred [69].

Parsimony approaches such as Clark’s algorithm [70] attempt to find the minimum number of unique haplotypes in a data set. The accuracy of this method depends on the assumption that markers are tightly linked and largely assignable to common haplotypes. Therefore, such algorithms over-predict common haplotypes. Coalescent-based methods and HMMs are also commonly employed to model population haplotype frequencies. The software programs *PHASE* [45], *fastPHASE* [41], *MaCH* [44], *IMPUTE2* [43], *SHAPEIT* [71], and *Beagle* [40] implement such methods (Table 2). These methods estimate parameters iteratively, so they work well with a small number of genetic markers residing on a short haplotype block.

*SHAPEIT* (*segmented haplotype estimation and imputation tool*) scales linearly with the number of variants, samples and conditional haplotypes used in each iteration and can be run efficiently on whole chromosomes [46,71]: it was applied to generate a haplotype map of 38 million SNPs for phase 1 of the 1000 Genomes Project [63,69]. This program is versatile for population-based studies as it is able to handle data from combinations of unrelated individuals, duos and trios. *SHAPEIT2* [51] adds the ability to incorporate molecular information from sequence reads, incorporating calls and base-quality scores in a probabilistic model. It works best for high-coverage sequence. O’Connell *et al*. [72] have incorporated *SHAPEIT2* into a general haplotyping workflow that can also detect state consistency errors in pedigrees [64].

Wang *et al*. [48] developed a population imputation pipeline, *SNPTools*, to phase low-coverage data obtained from phase 1 of the 1000 Genomes Project. *SNPTools* statistically models sequence data, scores polymorphic loci, and generates genotype likelihoods using a Binary sequence map (BAM)-specific Binomial Mixture Model (BBMM). The genotype likelihoods can then be integrated into *SNPTools*’ imputation algorithm or other algorithms such as *Beagle* to estimate phased genotypes and haplotypes. *SNPTools*’ haplotype imputation algorithm employs a four-state constrained HMM sampling scheme that assumes that the individual haplotype is a mosaic of the four parental haplotypes. *WinHAP* estimates multi-SNV haplotypes from large-scale genotype data [49]. This software simplifies the *2SNP* algorithm, using pairs of heterozygous SNVs to generate initial haplotypes and subsequently to construct a linear tree that makes it possible to infer a solution for the haplotype phase [73]. These haplotypes are then improved by applying scalable sliding windows. Last, parsimony is used to iteratively restrict the number of haplotypes.

The accuracy of population haplotyping can be improved by modeling population substructure and detecting cryptic relatedness. Such issues may be overcome by exploiting algorithms originally conceived for identical-by-descent (IBD) detection [74]. Programs such as *fastIBD* [40] and *GERMLINE* [18] leverage population level IBD to define haplotypes [75]. The extent of shared IBD between a pair of individuals depends on the number of generations since their last common ancestor as recombination and mutation increase genetic diversity. *GERMLINE* directly matches portions of haplotypes between individuals from phased genotype data. *FastIBD* uses a HMM approach for IBD detection of shared haplotypes from unphased genotype data. IBD segments are identified by modeling shared haplotype frequencies that account for background levels of LD.

Most of the available algorithms for population inference of haplotypes from WGS require careful balancing of computational speed and accuracy. They also rely on the availability of well-characterized, population-matched reference datasets [76]; these need to be large enough to sample rare variants. Population-based phasing methods are probabilistic, limited to generation of short haplotype blocks, and will incorrectly phase rare combinations of variants, exactly those combinations most likely to be medically important. Moreover, haplotypes derived from algorithms that include population inference are likely to have an error rate that is unacceptably high for medical purposes.

If an individual is a member of a completely characterized isolated population, the accuracy of population-based haplotypes can be very high. Such haplotyping has been demonstrated by Kong *et al*. [77] for the Icelandic population. Use of such databases in combination with methods to phase *de novo* mutations and haplotypes resulting from recent recombinations could both permit increased haplotype quality and reduce the need for genetic and molecular haplotyping in patients from these populations.

## Combination strategies

Combinations of molecular, genetic and population-based methods may work better than any single approach, by combining strengths and minimizing weaknesses (Table 2). *HARSH* evolved from *Hap-seqX*, combining haplotype assembly with a reference population dataset to predict haplotypes from WGS data [50,78]. *Prism*, mentioned earlier, is another recent hybrid algorithm [36]. *PPHS* (*Perfect Phylogeny Haplotypes from Sequencing*) is another combination approach that combines population and molecular analysis by using raw sequence data to construct a phylogenetic tree for every short region [52]. The phylogeny model assumes that there are no recurrent mutations or recombination within each short sequence region. For each set of SNPs in a given window, the algorithm reconstructs a local phylogenetic tree by expectation maximization and assigns haplotypes to individuals. The results for each window are then stitched together using the output of *Beagle* as a guide. Combination strategies such as these may increase the accuracy of population inference methods by leveraging the information provided by sequence data, or may supplement genetic analyses with population data, as described by Dewey *et al.* [66]. A combination of genetic, physical and population-based approaches in a quartet yielded complete genome phasing, including phasing of 99.8% of fully heterozygous variants [53].

## Clinical applications of whole-genome haplotyping

Local haplotyping has been and will remain important for genomic diagnostics. The immediate impact of whole-genome haplotyping will be to provide all local haplotypes. Local haplotypes are well known for the major histocompatibility complex (MHC) and several other loci, including the ApoE4 haplotype of the ApoE locus. MHC haplotypes are important for predicting graft compatibility and for prediction of the risks and protectivity of many phenotypes, notably type 1 diabetes [79]. In many cases, the causative variant is not known, and the observed haplotype serves as a proxy for assaying the unknown single variant that lies in or is linked to that haplotype. In other cases, such as ApoE4, multiple coding variants must lie on the same haplotype within a single coding sequence in order to effect a particular phenotypic change. Family-based haplotyping to identify compound heterozygosity as a cause of recessive Mendelian disease is also fairly routine. Fetal and newborn diagnostics will also benefit from haplotyping. Spearheading such an approach in 2012, Kitzman *et al*. [80] inferred haplotypes of a human fetus using haplotype-resolved genome sequencing of the mother, shotgun sequencing of the father, and deep sequencing of maternal plasma.

Pathogenic rare variants will be a significant source of concern when practicing genomic medicine. Thus, an important clinical application of haplotypes will occur at the largely unseen analysis stage - in improving variant calling and avoiding false alarms. Already, software tools such as *Platypus* (www.well.ox.ac.uk/platypus) are being developed to produce improved base calling as informed by haplotypes [63].

As knowledge and methods improve, understanding the functional interactions between regulatory elements and coding regions will permit medical decision-making that is based not only on the predicted effects of variants on the function of a protein, but also on combining separate predictions of (a) the functions of the two proteins produced by the two alleles of the encoding gene, and (b) the effects of the two *cis*-regulators of these two proteins. For example, if one of the *cis*-regulators markedly increases expression while the other decreases expression, and one protein is defective while the other is normal, then one combination of *cis*-regulators with the protein-coding alleles will produce wellness whereas the other combination will produce disease [4].

## Conclusions and future directions

High-throughput short-read sequencing enabled rapid advances following the HGP. Unfortunately, haplotyping got left by the wayside, as the long reads characteristic of the HGP gave way to cheaper short reads. Now a combination of new strategies and new technologies is enabling the determination of personal haplotypes that will soon be economical for more routine medical use. The new strategies that we have discussed enable the use of cheap short reads for inferring longer haplotypes, typically by physically or computationally placing these reads into haplotype bins. Some new technologies, such as Hi-C, facilitate this binning process, whereas other new technologies will enable the generation of cheaper long reads.

**Figure 3:**
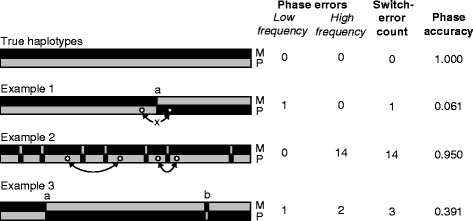
**Switch errors and quality metrics.** We present four scenarios involving low- and high-frequency switch errors and the resulting values for two quality metrics: total switch rate, and the pairwise metric 2_*_ *cse*-1, which we term the ‘phase accuracy’ (Box 3). The latter was computed on the assumption that each local ‘back and forth’ switch error affects 1% of the markers in the chromosome. The true haplotypes (maternal (M) and paternal (P)) are shown at the top. In Example 1, there is a single phase-error switch at ‘a’; variants on opposite sides of the error are incorrectly phased (denoted by the ‘x’ on the curved arrow). This situation might arise if a parent’s haplotype is inferred by subtraction of a child’s haplotype, missing a meiotic recombination. Example 1 thus has a low-frequency phase error but no high-frequency errors. In Example 2, there are many high-frequency errors, but no low-frequency errors. Most variant pairs are separated by even numbers of switch errors, and are thus properly phased (curved arrows). This error pattern might arise from a long-read technology such as strobe sequencing. In Example 3, there are three switch errors, one at ‘a’ and two at ‘b’. This haplotype could have arisen from a false haplotype assembly join at ‘a’ and a sequence error of a single base at ‘b’. Example 3 thus has a mix of low- and high-frequency errors. In terms of the pairwise metric, the single switch in Example 1 most strongly affects the long-range haplotype quality, while the several localized switch errors (each pair affecting just one or a few markers) degrade haplotype quality only modestly.

Considering the garbage-in-garbage-out principle, and that most current algorithms perform near perfectly on error-free data, improving sequencing error rate is probably the most critical factor for improving haplotypes [81]. In other words, to improve haplotypes for use in genomic medicine, a focus on phasing algorithms and methodologies is not necessarily the greatest requirement, but rather a focus on improving the input data. More phase errors can arise with whole-genome data than with genotyping chip data. The SNPs included in genotyping chips tend to be selected for Hardy-Weinberg equilibrium, and so any SNP with a heterozygote frequency that is unexpected in relation to the allele frequencies is excluded. Such pre-selection is not done for WGS data. The ability to phase WGS data can be confounded by reference sequence errors, reference gaps and centromeres, and long interspersed nuclear elements (LINEs) [8]. Methods are needed for filtering out these regions or for handling them in a probabilistic framework with appropriate confidence statistics. In conjunction with improvements in sequence quality, the generation of long sequence reads (of 10,000 bases or longer) is another key factor for haplotype improvement [18,19,82]. Reduction of sequencing error will have the greatest impact on high-frequency switch error, whereas improvements in read length will have a greater impact on low-frequency switch error (Boxes 1 and 3, and Figure 3). Because of the importance of compound heterozygote analysis and within-gene phasing for medical applications, high-frequency switch errors must be minimized. Commoditization of haplotyping will also be necessary, and as this occurs, the costs of the various approaches will become less opaque. Other commodity technologies, such as sequencing, are best performed in high-throughput operations because such facilities offer a concentration of expertise, economy of scale, standard operating procedures, and rigorous quality control. Clinical haplotype databases will need to be developed in parallel with haplotype commoditization, much like the ClinVar database for individual variants associated with disease [83].

It is now routine in medical genetics to consider compound heterozygosity in identifying disease risks and causes in patients. Typically, this search is either carried out by genetic haplotyping if the sequences of parents are available or is achieved by considering all possible haplotypes of detrimental variations within a gene. We identified compound heterozygosity causing Miller syndrome in our analysis of the first whole-genome sequenced family [64], but such analyses have been routine for years in the analysis of candidate genes, such as those for cystic fibrosis and breast cancer risk. Numerous examples include the identification of compound heterozygous causes of diseases, including the gene that encodes protein C in cerebral palsy [84], Charcot-Marie-Tooth neuropathy [85], and the gene encoding lysyl-tRNA synthetase in peripheral neuropathy [86]. Currently, much clinical screening for compound heterozygosity is done with exome sequencing, but we predict a shift towards WGS as costs drop. As the understanding and annotation of regulatory variants continues to improve, we will see an increasing number of reports of *cis*-acting regulatory elements that alter gene expression and cause disease. Examples that have already been reported include a mutation in a RET enhancer that increases Hirschsprung disease risk [87,88] as well as mutations that affect thalassemia, hemophilia, hypercholesterolemia, and pyruvate kinase deficiency [89]. Increasingly, the phase of these regulatory elements with respect to the coding variants will be part of routine diagnostics. For example, Ludlow *et al*. [90] described a patient with a mutation in the promoter of one allele of *GP1BB* (encoding platelet glycoprotein Ib beta) and a deletion of the other allele, which together resulted in Bernard-Soulier syndrome [90].

DNA diagnostics and prognostics that have clinical applications in oncology are expanding rapidly. For example, particular haplotypes of *GNMT* (encoding glycine N-methyltransferase) differently predispose individuals to prostate cancer [91]. In many oncological applications, genetic and population phasing is of little value because of the large number of somatic mutations that may be present in tumor cells. Molecular phasing will therefore be the primary tool in this area, and algorithms that allow for multiple ploidy states will be important in handling the complexities of tumor genomes; currently most haplotype assembly algorithms assume diploidy. The MHC/HLA (human leukocyte antigen) locus is the most important haplotype influencing disease; and an understanding of the value of MHC haplotypes is therefore nothing new [92,93]. It has traditionally been difficult to use molecular techniques that avoid low-frequency switch errors between genes of the MHC. Applications of some of the new long-range haplotyping techniques, particularly those capable of *de novo* assembly of regions of personal genomes within the MHC that are not in the reference genome, are likely to allow better utility of MHC typing for research, prognostics, diagnostics, and tissue transplantation [93]. The genomic medicine of the future will rely on accurately mining patient sequence data to identify disease, wellness and actionable genes [6]. Genomics must move beyond simple single allelic and genotypic tests of association and familial-segregation to explain phenotypes. At the simplest level, whether two detrimental variants that affect ‘the same gene’ lie in *cis* or in *trans* may spell the difference between a healthy carrier and a diseased compound heterozygote. The paradigm of medical understanding must be shifted from ‘the function of a gene in an individual’ to ‘the functions of each allele of each gene in an individual’. To achieve this, we must transform the conceptualization of the genome in the minds of both clinicians and researchers from one that contains 22 autosomes and two sex chromosomes to one that contains 44 autosomes and two sex chromosomes, each with its own haplotype. Individual genome sequencing is being applied at all stages of life, from preimplantation, prenatal and neonatal diagnosis, to ‘no phenotype’ personalized genomics [94]. Whole-genome haplotypes will improve the precision of personalized predictive, preventive and participatory medicine.

## Box 1. Glossary

**Base-quality score:** A measure of the accuracy of each individual nucleotide (‘base’) call determined by an automated sequencing platform for a DNA molecule. Specifically, this measure estimates the probability of error for each nucleotide called, enabling the discrimination of correct and incorrect nucleotide assignments in a DNA sequence across different sequencing platforms. As first defined by Ewing and Green, the quality score (*q*) assigned to a single base-call is *q* = -10 × log_10_(*p*), where *p* is the estimated error probability of that call.

**Chromosome haplotype:** The longest possible haplotype. The full sequence of a chromosome that an individual inherited from one parent, possibly altered later by somatic mutations.

**Completion criterion:** A target for the fraction of alleles determined by assaying a defined set of positions. For example, a haplotype may be 99.99% complete if the set of defined positions are those on a genotyping array of one million markers (for example, 999,900 called genotypes/1,000,000 SNPs), but that same haplotype reported for all variable positions of a genome that varies at only a third of these million sites but also at a million other sites would be only 25% complete (333,300/1,333,333 variable positions). Haplotyping methods make trade-offs among cost, accuracy, length and completeness.

**Genetic haplotyping:** The process of inferring the phasing of variants observed in ordered genotypes according to the principles of Mendelian segregation of alleles in pedigrees.

**Haplotype:** A set of co-inherited alleles occurring on a single strand of DNA.

**Haplotype assembly:** The computational process of ‘stitching’ together shorter, overlapping fragments of DNA sequences into a single, long haplotype tract. This stitching process relies on overlapping sequences containing one or more SNVs and requires a reference genome to map the reads.

**Haplotyping:** (also known as phasing) The process of determining haploid DNA sequences (haplotypes) from unordered (unphased) genotype data.

**High-frequency phase error:** Errors in the reconstruction of a haplotype resulting from mis-assignment of an isolated allele (single-site) in the DNA sequence.

**Identical-by-descent:** Identical copies of an allele or segment of DNA that two individuals have inherited from a shared common ancestor.

**Low-frequency phase errors:** Errors in the reconstruction of a haplotype resulting from mis-assignment of blocks of adjacent alleles in the DNA sequence.

**Molecular haplotyping:** The direct observation of alleles on a single molecule of DNA.

**Population-based haplotyping:** The process of assigning the most likely order of common alleles along each haploid segment of DNA according to the frequency of observation in a large sample set. This method constructs haplotypes from unordered genotype data.

**Quality metrics:** A system of standardized formal measures required to reliably quantify the accuracy and precision of a technique, which in the context of this review is the reconstruction of the true haplotype.

**Switch-error rate:** The proportion of heterozygous sites in each reconstructed haplotype that are mis-assigned relative to the heterozygous site that directly precedes it. It is also defined as one minus the switch accuracy.

## Box 2. Biological and medical importance of haplotypes

- Haplotypes can be used to study human migration, evolutionary selection and population structure [95-97]. They can be used for admixture mapping [98], imputation of regions lacking genotype information [44,99,100] and to improve the power of genetic association [101-104]. Haplotypes are critical for identifying identical-by-descent (IBD) regions that are shared between pairs of individuals [42,68,74].
- Haplotyping can aid the detection and correction of erroneous or missing sequencing data - for example, by detecting inconsistencies between the genotypes within a family [8,64]. Detecting and resolving such errors may prove crucial for medical interpretation of individual genomes, particularly when considering rare (or ‘private’) variants that affect the expression or function of medically important genes.
- Diploid haplotypes may display different functional profiles depending on the combination of functional elements [30]. Haplotypes are therefore essential for fundamental understanding of the roles of genetic regulation, epigenetic regulation, and chromatin modification in the human genome, and their phenotypic consequences. Haplotyping can enable the detection of compound heterozygosity, which is increasingly recognized as an important cause of genetic disease [64,84-86]. Functional *cis*-acting regulatory elements are known to alter gene expression and can cause disease [87-89]. Allele-specific expression and imprinting are also mediated by haplotypes [105,106]. Chromosome-length haplotypes are essential for assessing the functional consequences of distantly located variants and their interactions.
- Haplotyping is valuable in diagnosing loss of heterozygosity in cancer [91] and for establishing haplotypes of the major histocompatibility complex (MHC) that are important for autoimmunity and for organ transplantation [92,93].
- Haplotyping can determine the parental origins or occurrence of *de novo* mutations [107].

## Box 3. Quality metrics

Improvements in genome sequencing were enabled by the introduction of quality metrics such as completion criteria and base-quality scores [108,109]. Haplotyping typically uses relatively few formal metrics. Use of standard metrics for haplotyping will lead to algorithmic improvements, and will aid in deciding which algorithms should be used for particular purposes. The best method and metrics for haplotyping will depend on the application. For the purpose of assaying compound heterozygosity within a gene, switch errors (Box 1) between genes are irrelevant, but local switch errors are crucial. Conversely, for the purpose of detecting IBD blocks, a pair of adjacent local switch errors is inconsequential but long-range switch errors are crucial. Therefore the number of switch errors and their locations with respect to each other and the genome are important metrics.

The completeness of a haplotype reconstruction is also important, that is, how many positions are phased. Completeness statistics require that reported haplotypes include specification of the domain of positions under consideration, which might be the entire genome or could be restricted to commonly variable positions, positions observed to vary within a family, positions on a commercial genotyping panel, or a sparse set of markers such as short tandem repeats. Occasionally, the domain is chosen to be the set of all heterozygous markers in a data set (for example, [10]). Error metrics computed on this domain are useful for comparing multiple parameterizations of algorithmic approaches to the same data set, but have limited utility for comparisons between datasets. This is because the set of heterozygous positions is dependent on many factors, such as underlying quality of the reference genome, parameters in genotype-calling algorithms, data pre-processing that might eliminate poor-quality reads or genotype calls, and the amount and character of repetitive sequence in the targeted region or genome.

A common metric is the switch-error rate, sometimes known as phase error, switch accuracy, or recast (reformulated) as switch distance. Switch error is a tabulation of the number of times a reported haplotype ‘jumps the tracks’ between two true haplotypes [45,110,111]. On the basis of mother-father-offspring trio comparisons, the 1000 Genomes Project Consortium reported a switch error every 300 to 400 kb on average [63]. In its usual form, the switch-error rate is identical for a single base error (two switches very close together) or for two switches on different arms of a chromosome (two switches very far apart). If there is a single base error, the chromosomal haplotype is nearly perfect, but if the switches occur on different arms of a chromosome, the resulting haplotype is severely marred. In 2011, we introduced the concept of a smoothed switch error, and a declining relationship of switch error as a function of lowering the high-frequency cutoff [8]. Therefore, we can employ switch error in a continuous manner by varying resolution for the smoothing of switch errors, with a set of metrics analogous to those for sequence assembly [108]. Low-frequency switch errors represent phenomena that are different from those represented by high-frequency switch errors, both in terms of the utility of the data and in troubleshooting the causes of error (Figure 3).

The density and quality of markers both affect switch errors. For example, statistics can be skewed by reporting switch error across a small set of markers selected for the highest data quality. However, inclusion of all of the homozygous markers will increase the switch-error denominator and reduce the metric. Therefore, switch error is often reported on the basis of only those markers that are heterozygous in a particular analysis, leading to a high dependence on the particulars of SNP selection during either data acquisition or algorithm pre-processing. We recommend computing switch errors in conjunction with completeness metrics. Reasonable standards include the set of all positions in the reference genome, specific releases of the HapMap project, or particular commercial genotyping panels. Use of several different reference sets is important because not all markers are equivalently easy to phase. For example, common SNPs are more likely than rare SNVs to be heterozygous in all family members, at which point they become impossible to phase by genetic methods.

A metric that is related to switch error is the fraction of all pairs of markers in proper phase with each other (regardless of adjacency). Matsumoto and Kiryu [39] proposed a measure for haplotyping accuracy - the fraction of correctly phased pairs - that is based on the pairwise consistency of markers. We strongly endorse this metric, which we call the ‘complete switch error’ (*cse*), as it is robust to manipulation by parameterization and penalizes global effects more than local effects. We also recast *cse* specifically for evaluating a haplotype for compound heterozygosity by using it to calculate the excess fraction of the pairs of heterozygous variants in a particular gene that are correctly phased. This ‘phase accuracy’ metric is 2_*_cse-1 and represents the likelihood that any two heterozygous alleles in a diploid gene are correctly phased with respect each other. Perfect phasing produces a phase accuracy of 100%. A single switch error in the middle of a gene would drop phase accuracy to zero, as would a switch error between every pair of markers. With completely random phasing, half of all pairs of variants are correctly phased relative to each other, and thus the lowest likely operational value for phase accuracy is zero, although we note for the sake of mathematical robustness that with a very small number of variants, discrete effects could reduce the phase accuracy to below zero. Averaging phase accuracy across all genes encompassed by a study (for example, all known genes) produces a genome-wide metric for evaluating a haplotyping algorithm.

The exact choice of reference sequence is an additional parameter that affects haplotyping quality, particularly with respect to completion. The MHC locus, and in particular the HLA-DR region, is notorious for being poorly represented in current reference genomes. Therefore, if one is evaluating a haplotype across HLA-DR, the choice of a reference sequence that does not contain HLA-DR will falsely elevate phase-completeness statistics.

How can we know the error in a haplotype if we do not know the truth for certain? Frequently, molecular haplotyping will be the most accurate method. Genetic haplotyping is accurate but will have errors resulting from *de novo* mutations, and is particularly unreliable for cancer genomes. Population-based phasing is the least reliable approach, but offers the most probable phasing when other information is unavailable. Therefore, the best results are obtained by combination methods that integrate all available molecular-phasing evidence and genetic haplotyping where the samples are available, and supplement these with population inference as needed. Comparisons of different methods applied to the same genomes is time consuming and expensive but remains valuable *in lieu* of a gold-standard methodology, which is currently lacking for whole-genome haplotyping.

## Abbreviations

BAC: Bacterial artificial chromosome
BAM: Binary alignment map
BBMM: BAM-specific Binomial Mixture Model
CNV: Copy number variant
FACS: Fluorescence-activated cell sorting
*HASH*: Haplotype assembly for single human
HGP: Human Genome Project
HLA: Human leukocyte antigen
HMM: Hidden Markov model
IBD: Identical-by-descent
LD: Linkage disequilibrium
LFR: Long fragment read
LINE: Long interspersed nuclear element
MCMC: Markov chain Monte Carlo
MEC: Minimum error correction
MHC: Major histocompatibility complex
MIE: Mendelian inheritance error
*PPHS*: *Perfect Phylogeny Haplotypes from Sequencing*
SCE: State consistency error
SIH: Single individual haplotyping
SLRH: Statistically aided, long-read haplotyping
SNAP: SNP Annotation and Proxy Search
SNP: Single nucleotide polymorphism
SNV: Single nucleotide variant
WGS: Whole-genome sequencing
